# Preparation and Photochemistry of Hydroxy Isocyanate

**DOI:** 10.1021/acs.jpca.5c03245

**Published:** 2025-07-07

**Authors:** Guohai Deng, Caio M. Porto, Artur Mardyukov, Peter R. Schreiner

**Affiliations:** † Institute of Organic Chemistry, 9175 Justus Liebig University, Heinrich-Buff-Ring 17, 35392 Giessen, Germany

## Abstract

We describe the first spectroscopic identification of hitherto experimentally unreported hydroxy isocyanate HONCO, a potential candidate for interstellar medium and prebiotic chemistry. This planar chain molecule was prepared in the gas phase through flash vacuum pyrolysis of phenyl *N*-hydroxycarbamate at 650 °C and was subsequently trapped in argon matrices at 3.5 K. Its characterization was accomplished by means of matrix isolation IR and UV/vis spectroscopy together with quantum chemical computations. Upon UV light (λ = 313 nm) irradiation, HONCO decomposes into hydrogen-bonded complexes of HON and HNO with CO.

## Graphical abstract

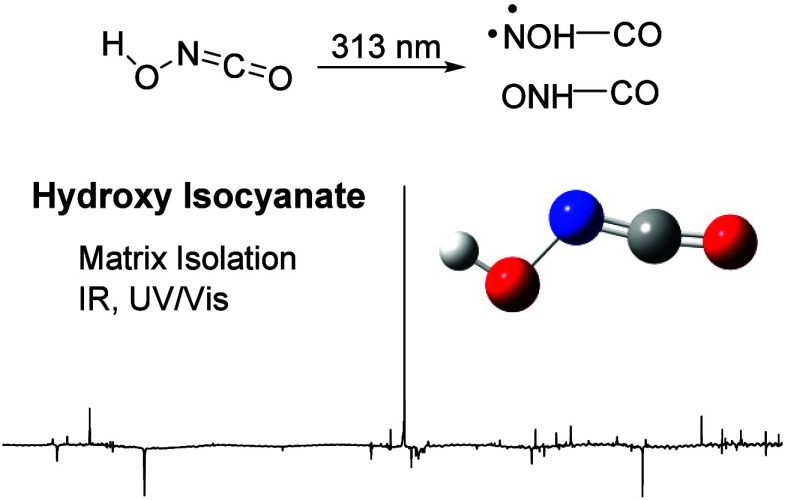

## Introduction

Isocyanates (R–N=C=O) play a critical role not only in the synthesis of biologically active heterocycles,^1^ the fabrication of polyurethane materials,^2^ and the degradation of pesticides^3^ but also in astrochemistry and even prebiotic chemistry. The latter originates from their role in the synthesis of amino acids, in the polymerization of peptides,^4^ and in the production of nucleotides^5^ as well as nucleosides.^6^ To date, only five isocyanate derivatives have been detected in the interstellar medium (ISM): the •NCO radical (**4**),^7^ which is the simplest molecule containing a peptide bond backbone; isocyanic acid HNCO (**5**),^8^ the first detected isocyanate species in space; N-protonated isocyanic acid H_2_NCO^+^ (**6**);^7,9^ methyl isocyanate CH_3_NCO (**7**)^10–13^ and ethyl isocyanate CH_3_CH_2_NCO (**8**).^14^ Hence, the fundamental properties such as structures, spectral data, and photochemistry of simple isocyanates have been the focus of numerous experimental and theoretical investigations.^15–19^

Hitherto experimentally unreported parent HONCO (**1**) is implied in the combustion of organic compounds and atmospheric reactions^20,21^ and can be regarded an interstellar molecular candidate formed from the combination of the interstellar species HO^22^ and NCO.^7^ Theoretical studies indicate that **1** might also form in the oxidation of HCN/HNC in oxygen-rich gaseous mixtures or via the CH + NO_2_, NH + CO_2_, N + HOCO, and HCO + NO reactions.^23–27^ A recent *ab initio* study at the CCSD­(T)/CBS level of theory on the stationary structures of the HCNO_2_ stoichiometry found that chain-like, planar **1** is the most stable species among 20 identified minima.^28^ Hence, while the molecular structure and spectroscopic properties of **1** have been computationally studied well,^29^ **1** has not been identified experimentally. Milligan and co-workers proposed **1** as a quasi-linear chain molecule generated from the UV–vis light photolysis of HN_3_ in CO_2_ matrixes at low temperatures.^30^ However, only the next higher-lying HNCO_2_ isomer, HNC­(O)­O, was spectroscopically identified. This was subsequently explained by computations of the S_0_ and S_1_ states of **1**, which indicate that it is unstable under UV/vis light irradiation.^29^ Intermediate **1** (and its higher congeners with H being replaced by an aryl or alkyl group) was suggested in the synthesis of hydroxylamine-derived heterocycles.^31^ Here we report the first preparation and spectroscopic characterization of **1** and outline its unreported photoreactivity (see Scheme 1).

**Scheme 1:**
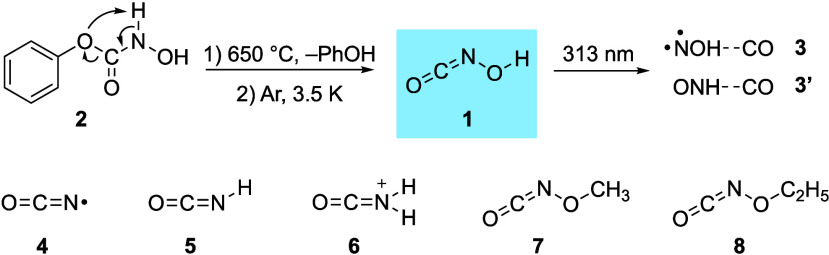
Hydroxy Isocyanate HONCO (**1**) Generated from Phenyl *N*-Hydroxycarbamate (**2**) through Pyrolysis and Trapping in an Argon Matrix and Subsequent Photoisomerization to **3**, **3′**; Structures of Isocyanates **4**–**8** Detected in the Interstellar Medium (ISM)

## Methods

### Experimental Methods

For the matrix isolation studies, we used an RDK 408D2 closed-cycle refrigerator cold head and an F-70 compressor system equipped with an inner polished CsI window for IR measurements. IR spectra were recorded between 7000 and 350 cm^–1^ with a resolution of 0.7 cm^–1^ with a Bruker Vertex 70 FTIR spectrometer and UV/vis spectra were recorded with a JASCO V-670 spectrophotometer equipped with an inner sapphire window. A high-pressure-mercury lamp equipped with a monochromator (LOT Quantum Design) or a low-pressure-mercury lamp (Gräntzel) fitted with a Vycor filter were used for irradiation.

For the high-vacuum flash pyrolysis experiment, we used a home-built, water-cooled oven, which was directly connected to the vacuum shroud of the cryostat. The pyrolysis zone consisted of a heatable 90 mm long quartz tube with an inner diameter of 7 mm, monitored by a Ni/CrNi thermocouple. The travel distance of the sample from the pyrolysis zone to the matrix was ∼45 mm. Phenyl *N*-hydroxycarbamate (**2**) (abcr) was evaporated from a Schlenk tube at 85 °C into a quartz pyrolysis tube. All pyrolysis products were co-condensed with a large excess of argon (typically 100–120 mbar from a 2000 mL storage bulb) on both sides of the matrix window at a rate of ∼1 mbar min^–1^, based on the pressure inside the Ar balloon. Pyrolyses were carried out at 650 °C. D_2_O was mixed with PhOC­(O)­N­(H)­OH to obtain PhOC­(O)­N­(D)­OH, PhOC­(O)­N­(H)­OD, and PhOC­(O)­N­(D)­OD and excessive D_2_O was removed from the mixture under reduced pressure.

### Computational Methods

Initial geometry optimizations were performed with Gaussian 16, Revision C.01 ^32^ at the B3LYP/def2-TZVP level of theory.^33–35^ Coupled cluster computations with single, double, and perturbative included triple substitutions, CCSD­(T),^36–38^ were carried out using the Dunning correlation consistent split valence basis sets cc-pVTZ^39^ and aug-cc-pVTZ^40^ with the CFOUR software package.^41^ The dominant excitation of the lowest computed state of **1** was >99%. Additionally, the T1 diagnostic and the largest T2 amplitude were computed to be 0.0184 and −0.0703, respectively, both from the CCSD­(T) computations. Taken together, these results strongly suggest that the ground state and low-lying excited states are well-described within a single-reference framework.

## Results and Discussion

The title molecule **1** was synthesized by flash vacuum pyrolysis (FVP) of phenyl *N*-hydroxycarbamate (**2**) at 650 °C. Specifically, **2** was evaporated at 85 °C and subjected to pyrolysis in a quartz tube. The pyrolysis products were mixed with excess argon before being condensed onto a cold matrix window at a temperature of 3.5 K.

The infrared (IR) spectrum of the pyrolysis products and the spectral changes upon irradiation in an Ar matrix are shown in Figure 1 (Figure S1 shows the raw IR spectrum of the pyrolysis of **2** in the Supporting Information). The decomposition of **2** is evident by the observation of phenol^42^ (b, 3639/3634, 1504/1501, 1176, and 689/687 cm^–1^) among the pyrolysis products. Besides the strong bands of CO_2_ (c, 2345.3 and 663.6 cm^–1^)^43^ and HNCO (d, 3516.8, 3505.7, 2259.0, 769.8, and 573.7 cm^–1^),^44^ and the weak bands of **2**, CO (e, 2138.7 cm^–1^)^45^ and NO (f, 1872.2 cm^–1^),^46^ we detected a new product (**1**, Figure S1) with IR bands at 3610.3, 2196.9, 1449.4, 1246.1, 862.5, 686.2, and 516 cm^–1^. The experimentally observed band positions are in remarkable concordance with the CCSD­(T)/cc-pVTZ computed anharmonic IR frequencies at 3651.3, 2259.0, 1441.5, 1241.6, 862.5, 686.3, and 515.6 cm^–1^ for the remaining product **1** (Table 1). In particular, the strongest band at 2196.9 cm^–1^ can be assigned to the NCO antisymmetric stretching mode, which is very close to that in CH_3_ONCO (2196.9 cm^–1^, Ar matrix)^47^ and PhONCO (2200.6 cm^–1^, Ne matrix; 2197.6 cm^–1^, Ar matrix).^48^ Another strong band at 3610.3 cm^–1^ belongs to the characteristic stretching vibration of the OH moiety in **1**, which is blue-shifted compared to that in *anti*-*syn* HONSO at 3548.6 cm^–1^ (N_2_ matrix).^49^ The assignments of these bands were also supported by the *d*-substitution experiment based on the characteristic isotopic shifts. For example, the stretching vibration of the OH groups in *d*-**1**, at 2669.0 cm^–1^, was red-shifted by 941.3 cm^–1^ (calc: 953.4 cm^–1^). The intense absorption band at 2196.9 cm^–1^ exhibited a red shift of 6.9 cm^–1^ (CCSD­(T)/cc-pVTZ: harmonic, 0 cm^–1^; anharmonic, 4.5 cm^–1^) in *d*-**1**. The combination band at 2158.3 cm^–1^ (calc: 1331.4 + 851.9 cm^–1^) was also found for *d*-**1**. The good agreement between the CCSD­(T)/cc-pVTZ anharmonically computed and experimentally measured frequencies of the **1** and *d*-**1** isotopologues strongly supports the successful preparation of **1** and *d*-**1** (Figure 1 and Table 1).

**Figure 1:**
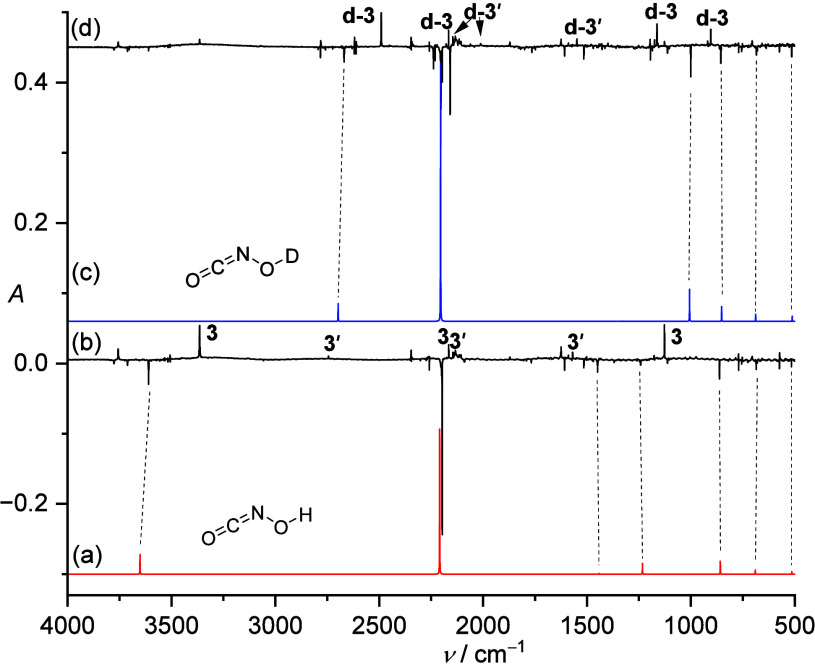
IR spectra showing the pyrolysis products of **2** with subsequent trapping in an argon matrix at 3.5 K. a) IR spectrum of **1** computed at CCSD­(T)/cc-pVTZ (anharmonic). b) IR difference spectra showing the photochemistry of **1** after irradiation with λ = 313 nm in argon at 3.5 K. Downward bands assigned to **1** disappear while upward bands assigned to **3** and **3**′ appear after 20 min irradiation. c) IR spectrum of *d*-**1** computed at CCSD­(T)/cc-pVTZ (anharmonic). d) IR difference spectra showing the photochemistry of *d*-**1** after irradiation with λ = 313 nm in argon at 3.5 K. Bands pointing downward assigned to *d*-**1** disappear and bands pointing upward assigned *d*-**3** and *d*-**3′** after 20 min irradiation.

**Table 1:** Experimentally Observed and Computed IR Frequencies of **1** and *d*-**1** at the CCSD­(T)/cc-pVTZ Level Using Second-Order Vibrational Perturbation Theory (VPT2) (Band Origins (cm^–1^) and Computed Intensities (km mol^–1^) in Parentheses)

	1			*d*-**1**			
Mode	Harmonic	Anharmonic	Ar, 3.5 K	Harmonic	Anharmonic	Ar, 3.5 K	Approx Assignment
9 (A′)	3841.6 (94)	3651.3 (71)	3610.3 (30)	2798.9 (43)	2697.7 (36)	2669.0 (17)	OH str
8 (A′)	2253.4 (623)	2209.0 (516)	2196.9 (100)	2253.3 (623)	2204.5 (769)	2190.0 (100)	NCO asym str
7 (A′)	1493.2 (56)	1441.5 (3)	1449.4 (12)	1365.0 (<1)	1331.4 (<1)	–^a^	HON bend
6 (A′)	1269.5 (48)	1233.0 (38)	1241.6 (7)	1027.5 (78)	1006.3 (185)	1000.7 (25)	NO str + HON bend.
5 (A′)	883.1 (51)	858.2 (46)	862.5 (14)	874.3 (36)	851.9 (35)	855.6 (8)	NO str
4 (A′)	694.6 (17)	690.0 (14)	686.3 (6)	692.6 (16)	688.2 (15)	684.6 (6)	NCO bend.
3 (A′)	514.3 (9)	514.1 (9)	515.6 (3)	513.1 (12)	512.2 (12)	514.7 (5)	NOC bend.
2 (A″)	226.2 (8)	217.7 (9)	–^a^	221.6 (8)	214.5 (9)	–^a^	
1 (A″)	204.9 (127)	171.1 (113)	–^a^	150.9 (65)	134.7 (64)	–^a^	

a Not observed.

Irradiation of the pyrolysis products in argon at 313 nm results in about 90% decomposition of HONCO after 30 min and the formation of two new groups of IR bands. Interestingly, HNC­(O)­O, HC­(O)­NO, and HN­(O)­CO) were *not* identified.^29^ Considering the previously reported photochemistry of covalent isocyanates^50^ and more recently the novel phosphinyl radical HPNCO• conversion to •NPH···CO and •PNH···CO complexes upon UV light irradiation,^51^ six possible products with H-bonding interactions were investigated computationally at B3LYP-D3­(BJ)/def2-TZVP (Figure S2).

Two groups of bands at 3364.4/2165.4/1127.2 cm^–1^ (**3**) and 2744.1/2145.6/1568.9 cm^–1^ (**3′**) are assigned to NOH···CO in the triplet ground state and ONH···CO in the singlet ground state, respectively, by comparison with the computed IR frequencies (Table S1 and Table S2). Both complexes have *C*_*s*_ symmetry. For product **3**, the strong band at 3364.4 cm^–1^ with an isotopic shift of −874.1 cm^–1^ (calc.: −942 cm^–1^) can readily be assigned to the H–O stretching mode, which is red-shifted compared to free HON (3467.2 cm^–1^, Ar matrix).^52^ For product **3′**, the first band at 2744.1 cm^–1^ shows an H/D isotope shift of −732.1 cm^–1^ (calc.: −766 cm^–1^) that is attributed to the H–N vibrational mode. This band is very close to the same vibrational mode of free HNO (2715.1 cm^–1^, Ar matrix).^52^ The CO vibrations in both complexes (2165.4 cm^–1^, **3**; 2145.6 cm^–1^, **3**′) are blue-shifted in comparison to the band at 2138.5 cm^–1^ for free CO in the same Ar matrix, and they show very small H/D isotopic shifts (+0.6 cm^–1^, **3**; +0.3 cm^–1^, **3**′). Similarly small blue shifts of the CO vibration were also observed for •NPH···CO, •PNH···CO and H_2_O_2_···CO.^51^ The two sets of IR bands for the characteristic HO/CO and HN/CO stretching modes and the H/D isotopic shifts as well as the computed frequencies (Table S1 and Table S2) strongly suggest assignment to the NOH···CO and ONH···CO complexes. The formation of these is due to the matrix effect that the photofragments HON/HNO and CO can not readily escape from the rigid Ar matrix cage.^51,53^ Similar arguments apply to the fact that we did not observe the dissociation of **1** into HO• and •NCO, because the barrier for radical–radical recombination will be small relative to the high exothermicity of this reaction. Second, the two radicals cannot escape the matrix cage and therefore would recombine very rapidly upon ending the irradiation. Attempts to convert NOH···CO into ONH···CO failed.

Along with the IR analysis, we carried out UV/vis experiments for the characterization of **1** (Figure 2). The UV/vis spectrum of matrix-isolated **1** displays a broad absorption band at λ_max_ = 203 nm. Consistent with the IR observation, the intensity of the 203 nm band decreases when irradiated at 313 nm. The broad band at 203 nm correlates well with the computed value at 202 nm (*f* = 0.031) at the TD-B3LYP/def2-TZVP level of theory. This band arises from a HOMO → LUMO+4 excitation, which correlates with a π → π* transition (Figure 2).

**Figure 2:**
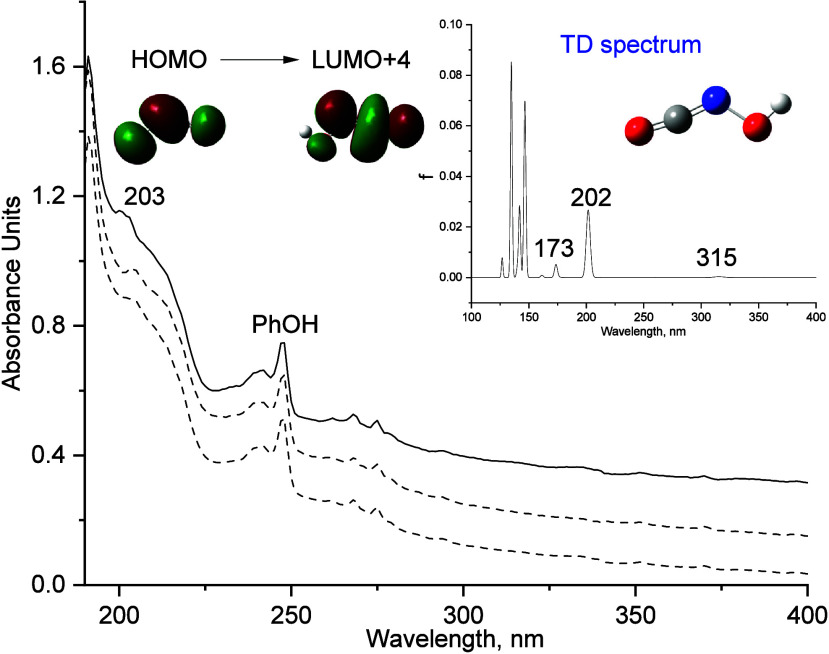
Solid line: UV/vis spectrum of **1** trapped at 10 K in Ar. Dashed lines: After irradiation of **1** at λ = 313 nm for 15 and 45 min in argon at 10 K. Inset: TD-B3LYP/def2-TZVP computed the electronic transitions for **1**.

The molecular structures of **1** and the two hydrogen-bonded complexes **3** and **3**′ computed at B3LYP/def2-TZVP are shown in Figure 3 and Figure S2, and these results are consistent with previous theoretical reports on the structures of **1** and its isomers. According to these, planar *C*_*s*_-**1** has a singlet ground state with a nearly linear N=C=O fragment. The second most stable isomer, HNC­(O)­O, lies +10.3 kcal mol^–1^ above **1** at B3LYP/def2-TZVP [5.5 kcal mol^–1^ at CCSD­(T)/CBS].^28^

**Figure 3:**
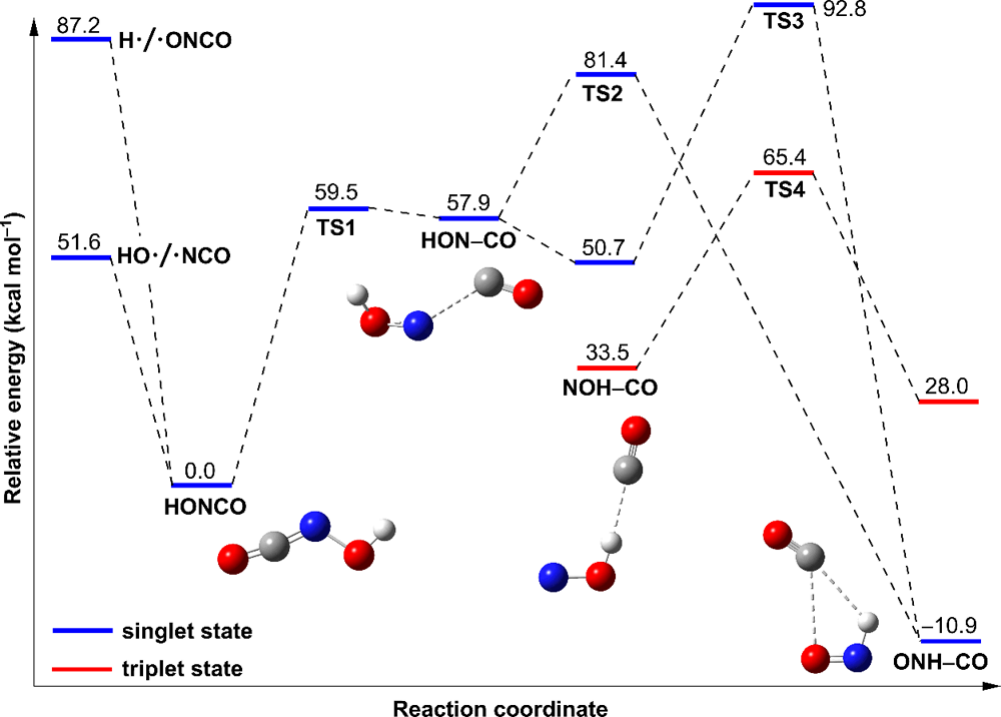
Computed potential energy profile for the decomposition of HONCO (**1**) at CCSD­(T)/aug-cc-pVTZ//B3LYP-D3­(BJ)/def2-TZVP. The energies are in kcal mol^–1^ and are ZPVE corrected, i.e., Δ*H*_0_.

As to the two hydrogen-bonded complexes, *C*_*s*_-**3** has an A″ triplet ground state with symmetry, while *C*_s_-**3**′ possesses an A′ singlet ground state. The computed singlet–triplet energy separation Δ­(*E*_ST_) of *C*_*s*_-**3** is +25.0 kcal mol^–1^ at CCSD­(T)/aug-cc-pVTZ//B3LYP-D3­(BJ)/def2-TZVP, while that of *C*_*s*_-**3′** is −38.9 kcal mol^–1^. The dissociation energy (*D*_e_) of *C*_*s*_-**3** is 2.8 kcal mol^–1^, about twice that of **3**′ (1.3 kcal mol^–1^) (Figure S2). The noncovalent hydrogen bond length in **3** (2.099 Å) is significantly shorter than that in •NPH···CO (2.438 Å) resulting in a smaller *D*_e_ of 1.5 kcal mol^–1^ for the latter.^51^ The stronger H-bond in *C*_*s*_-**3** also accounts for the larger blue shift (+26.9 cm^–1^) of the CO stretching frequency as compared to that in •NPH···CO (+5.5 cm^–1^) relative to free CO.^51^ In less stable *C*_*s*_-**3**′, the intermolecular hydrogen bond length is 2.500 Å, consistent with *C*_*s*_-**3**′ having a lower dissociation energy. In both complexes, the carbon atom of the CO moiety serves as the hydrogen bond acceptor. Such complexes have also been observed with water^54^ and ammonia.^55^

To better understand the photochemistry of **1**, the potential energy profile for the decomposition of **1** was explored computationally at the CCSD­(T)/aug-cc-pVTZ//B3LYP-D3­(BJ)/def2-TZVP level of theory (Figure 3). In line with the thermal persistence of **1** in the gas phase, we computed two barriers for its highly endothermic dissociation to HO•/•NCO and H•/•ONCO. The absence of •NCO and •ONCO is in accordance with the high bond dissociation energies for the O–N (51.6 kcal mol^–1^) and H–O (87.2 kcal mol^–1^) bonds of **1**, and the matrix cage effects as noted above. The activation barrier (**TS1**) for the CO-elimination to give the high-lying HON···CO complex is 59.5 kcal mol^–1^. The initially generated HON···CO complex is barely stable and will likely further isomerize to singlet *C*_*s*_-**3** via a barrierless transition state; this reaction is exothermic by 7.2 kcal mol^–1^. The second process refers to the intramolecular rearrangement from *C*_*s*_-**3** to *C*_*s*_-**3**′, which needs to surmount a barrier of 23.5 kcal mol^–1^. The overall reaction giving *C*_*s*_-**3**′ is exothermic by −10.9 kcal mol^–1^.

## Conclusions

We describe the first preparation and IR as well as UV/vis spectroscopic characterization of hydroxy isocyanate HONCO, a potential candidate molecule in the interstellar medium relevant to prebiotic chemistry. The firm spectroscopic evidence of HONCO is important in the context of the abiotic formation of amino acids and peptides for which isocyanates are likely to be important intermediates. Our studies aid the identification of HONCO in extraterrestrial environments.

## Supplementary Material



## Notes

Experimental infrared spectra, computed structures, vibrational frequencies, and atomic coordinates and energies (PDF)
