# Supplementary material for: Preparation and Photochemistry of Hydroxy Isocyanate
Source: J Phys Chem A. 2025 Jul 7;129(28):6350–5. doi: 10.1021/acs.jpca.5c03245 (PMC12278247; doi:10.1021/acs.jpca.5c03245)
Supplement: Supplementary file 1 [file jp5c03245_si_001.pdf]

Supporting Information

Preparation and Photochemistry of Hydroxy Isocyanate

Guohai Deng,[1] Caio M. Porto,[1] Artur Mardyukov,[1] and Peter R. Schreiner[1*]

[1]Institute of Organic Chemistry, Justus Liebig University, Heinrich-Buff-Ring 17, 35392 Giessen, Germany.

Table of Contents

Table of Contents.. S2

Figure S1 .... S3

Figure S2 .... S4

Table S1 .... S5

Table S2 .... S5

Optimized structures.. S6

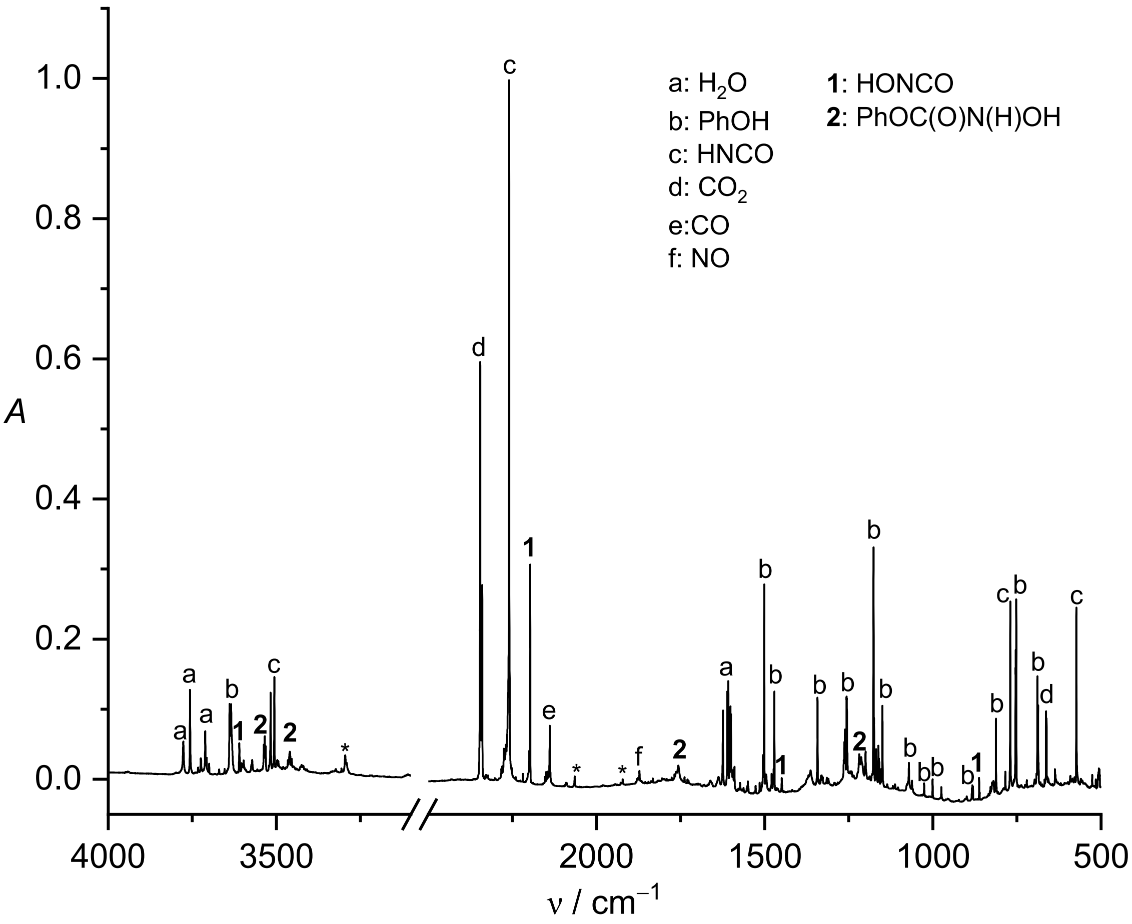

Figure S1. IR spectrum showing the product of pyrolysis of **2** with subsequent trapping in an argon matrix at 12 K. Unknown species are marked with *.

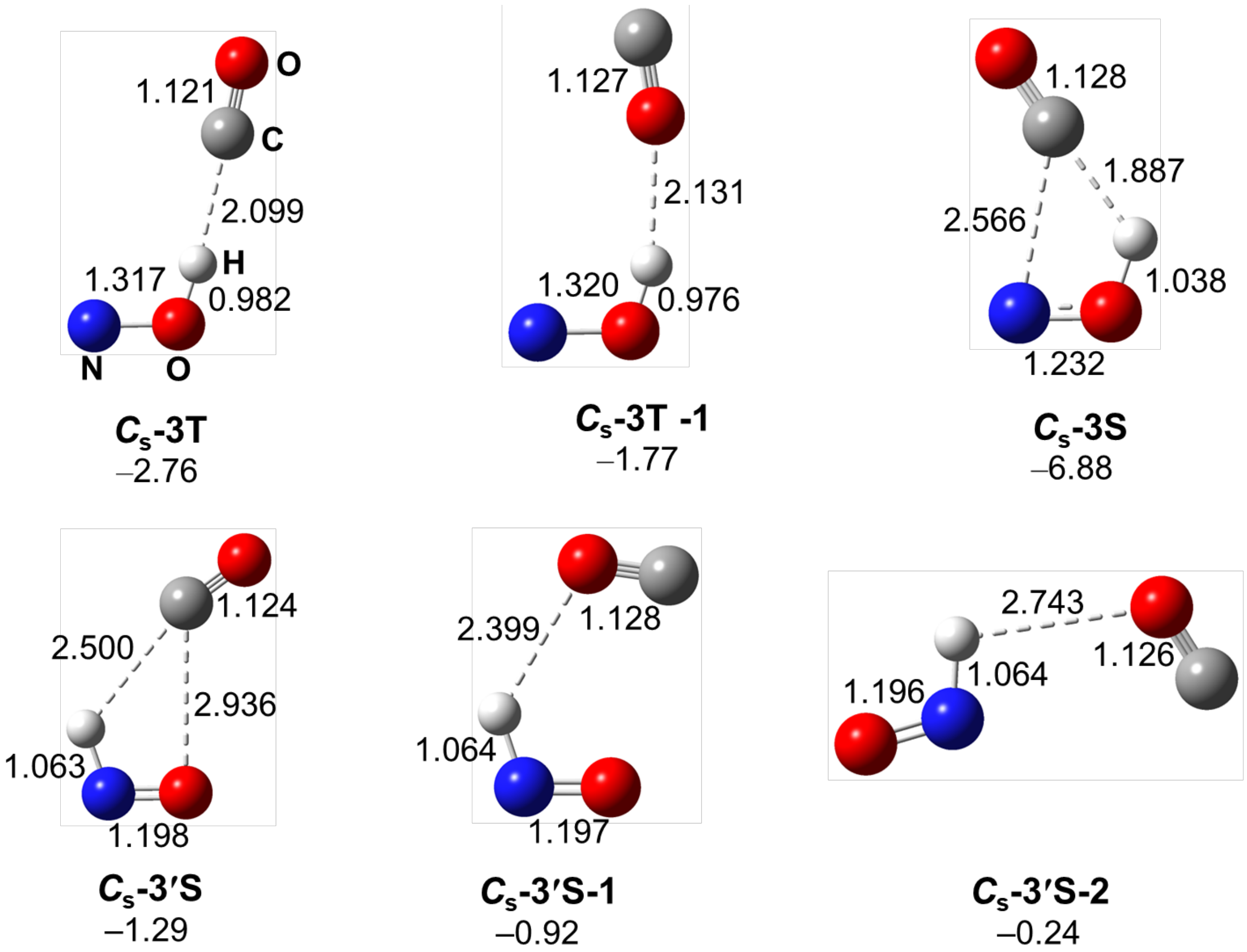

Figure S2. Computed structures (bond lengths in Å) and binding energies (ΔH_0 in kcal mol^{-1}) for the complexes at the B3LYP-D3(BJ)/def2-TZVP level of theory.

Table S1. Experimentally observed and computed IR frequencies of **3** and d-**3**, band origins in cm^{-1}, computed intensities (km mol^{-1}) in parentheses.

Mode	**3** computed [a]	**3** Ar, 3.5 K[b]	d-**3** computed [a]	d-**3** Ar, 3.5 K[b]	Assignment
9 (A′)	3487 (540)	3364.4	2545 (274)	2490.3	OH str.
8 (A′)	2248 (69)	2165.4	2248 (73)	2166.0	CO str.
7 (A′)	1280 (<1)	–	1205 (93)	1163.1	ON str.
6 (A′)	1169 (157)	1127.2	922 (41)	904.1	OH bend.
5 (A″)	418 (87)	–	310(41)	–	
4 (A′)	168 (8)	–	164 (7)	–	
3 (A′)	133 (2)	–	131 (2)	–	
2 (A″)	87 (9)	–	85 (9)	–	
1 (A′)	49 (6)	–	48 (6)	–	

[a] B3LYP(D3)/def2-TZVP, harmonic approximation, unscaled frequencies, intensities (in parentheses) in km mol^{-1}. [b] not observed.

Table S2. Experimentally observed and computed IR frequencies of **3′** and d-**3′**, band origins in cm^{-1}, computed intensities (km mol^{-1}) in parentheses.

Mode	**3′** computed [a]	**3′** Ar, 3.5 K[b]	d-**3′** computed [a]	d-**3′** Ar, 3.5 K[b]	Assignment
9 (A′)	2872 (68)	2744.1	2106 (36)	2012.0	NH str.
8 (A′)	2221 (92)	2145.6	2221 (93)	2145.9	CO str.
7 (A′)	1676 (64)	–	1667 (56)	–	NO str.
6 (A′)	1570 (28)	1568.9	1195 (13)	–	NH bend.
5 (A″)	292 (77)	–	220 (38)	–	
4 (A′)	203 (20)	–	193 (17)	–	
3 (A′)	121 (<1)	–	118 (<1)	–	
2 (A″)	66 (<1)	–	66 (<1)	–	
1 (A′)	57 (3)	–	57 (3)	–	

[a] B3LYP(D3)/def2-TZVP, harmonic approximation, unscaled frequencies, intensities (in parentheses) in km mol^{-1}. [b] not observed.

Computed atomic coordinates and energies of species for optimized structures.

1

B3LYP/def2-TZVP

0 1

H	2.32452100	0.25404500	0.00001100
C	-0.65903100	0.07118900	-0.00000200
O	-1.79691000	-0.17599700	-0.00000900
N	0.47955300	0.51725200	0.00000500
O	1.58100900	-0.36174600	0.00000400

Zero-point correction= 0.026109

Thermal correction to Energy= 0.030336

Thermal correction to Enthalpy= 0.031280

Thermal correction to Gibbs Free Energy= 0.000103

Sum of electronic and zero-point Energies= -243.905128

Sum of electronic and thermal Energies= -243.900901

Sum of electronic and thermal Enthalpies= -243.899957

Sum of electronic and thermal Free Energies= -243.931134

1

CCSD(T)/cc-pVTZ

0 1

O	0.00000000	0.00000000	0.00000000
H	0.00000000	0.00000000	0.96229311
C	1.75181938	0.00000000	-1.44421561
N	1.39601000	-0.00001234	-0.26005818
O	2.27458712	0.00000832	-2.49131839

Zero-point correction= 0.02559936

Sum of electronic Energies= -243.47819309

3-singlet

B3LYP-D3(BJ)/def2-TZVP

0 1

H	-0.71612700	-0.99686100	0.00005600
C	1.02670400	-0.27454200	0.00007400
O	2.07997000	0.12812900	0.00008800
N	-1.32609700	0.74861200	-0.00013200
O	-1.60014700	-0.45265000	-0.00003600

Zero-point correction= 0.021274

Thermal correction to Energy= 0.026438

Thermal correction to Enthalpy= 0.027382

Thermal correction to Gibbs Free Energy= -0.006730

Sum of electronic and zero-point Energies= -243.819750

Sum of electronic and thermal Energies= -243.814586

Sum of electronic and thermal Enthalpies= -243.813642

Sum of electronic and thermal Free Energies= -243.847754

3-triplet

B3LYP-D3(BJ)/def2-TZVP

0 3

H	0.00000000	0.79614600	0.00000000
C	-0.92044500	-1.09070300	0.00000000
O	-1.38856300	-2.10971000	0.00000000
N	1.79561100	1.36131600	0.00000000
O	0.50773700	1.63706800	0.00000000

Zero-point correction= 0.020588

Thermal correction to Energy= 0.026628

Thermal correction to Enthalpy= 0.027572

Thermal correction to Gibbs Free Energy= -0.010515

Sum of electronic and zero-point Energies= -243.846791

Sum of electronic and thermal Energies= -243.840751

Sum of electronic and thermal Enthalpies= -243.839806

Sum of electronic and thermal Free Energies= -243.877893

3′-singlet

B3LYP-D3(BJ)/def2-TZVP

0 1

O	-1.43798800	-0.65386600	0.00000000
H	-1.08695600	1.14576400	0.00000000
C	1.30052700	0.40403100	-0.00000100
N	-1.89351600	0.45367000	0.00000100
O	2.25528900	-0.18933900	0.00000100

Zero-point correction= 0.020679

Thermal correction to Energy= 0.026823

Thermal correction to Enthalpy= 0.027768

Thermal correction to Gibbs Free Energy= -0.009439

Sum of electronic and zero-point Energies= -243.877509

Sum of electronic and thermal Energies= -243.871365

Sum of electronic and thermal Enthalpies= -243.870421

Sum of electronic and thermal Free Energies= -243.907627

3′-triplet

B3LYP-D3(BJ)/def2-TZVP

0 3

O	-2.42567500	-0.43077600	-0.00002000
H	-0.64235600	0.36812000	-0.00366700
C	1.62973200	0.05688300	-0.00271600
N	-1.66283300	0.52496000	0.00101600
O	2.73864900	-0.11724200	0.00162700

Zero-point correction= 0.020077
Thermal correction to Energy= 0.026434
Thermal correction to Enthalpy= 0.027378
Thermal correction to Gibbs Free Energy= -0.011921
Sum of electronic and zero-point Energies= -243.861930
Sum of electronic and thermal Energies= -243.855573
Sum of electronic and thermal Enthalpies= -243.854629
Sum of electronic and thermal Free Energies= -243.893928

HON-CO

B3LYP-D3(BJ)/def2-TZVP

0 1

O	1.99798500	-0.22985700	-0.15734900
H	2.42042500	-0.57392300	0.68207500
C	-1.30892700	-0.46339000	0.05809900
N	1.05776600	0.56583200	0.07927400
O	-2.24438800	0.15403700	-0.04084900

Zero-point correction= 0.019942
Thermal correction to Energy= 0.026197
Thermal correction to Enthalpy= 0.027141
Thermal correction to Gibbs Free Energy= -0.010704
Sum of electronic and zero-point Energies= -243.811484
Sum of electronic and thermal Energies= -243.805230
Sum of electronic and thermal Enthalpies= -243.804286
Sum of electronic and thermal Free Energies= -243.842131

TS1

B3LYP-D3(BJ)/def2-TZVP

0 1

O	1.89953100	-0.21651000	-0.14794200
H	2.31680800	-0.51184300	0.70149500

C	-1.13993500	-0.41247600	0.04713200
N	0.91576900	0.54926700	0.06418000
O	-2.13547900	0.10923900	-0.03125100

Zero-point correction= 0.020812
Thermal correction to Energy= 0.025791
Thermal correction to Enthalpy= 0.026735
Thermal correction to Gibbs Free Energy= -0.007157
Sum of electronic and zero-point Energies= -243.808304
Sum of electronic and thermal Energies= -243.803325
Sum of electronic and thermal Enthalpies= -243.802381
Sum of electronic and thermal Free Energies= -243.836273

TS2

B3LYP-D3(BJ)/def2-TZVP

0 1

O	2.13674300	-0.24752300	-0.07415800
H	2.20685500	-0.79221800	0.76011300
C	-1.49599400	-0.50038700	-0.12178200
N	1.22036200	0.60304700	-0.00691200
O	-2.35842100	0.19417400	0.07652800

Zero-point correction= 0.019271
Thermal correction to Energy= 0.025145
Thermal correction to Enthalpy= 0.026089
Thermal correction to Gibbs Free Energy= -0.011456
Sum of electronic and zero-point Energies= -243.810212
Sum of electronic and thermal Energies= -243.804339
Sum of electronic and thermal Enthalpies= -243.803394
Sum of electronic and thermal Free Energies= -243.840939

TS3

B3LYP-D3(BJ)/def2-TZVP

0 1

O	2.20039800	-0.36069100	-0.19281900
H	2.12469000	0.01161500	0.85012200
C	-1.55425800	-0.35246100	0.30272500
N	1.31034400	0.57962300	0.06315500
O	-2.44684200	0.11641500	-0.19575100

Zero-point correction= 0.015222

Thermal correction to Energy= 0.021949

Thermal correction to Enthalpy= 0.022894

Thermal correction to Gibbs Free Energy= -0.017270

Sum of electronic and zero-point Energies= -243.757159

Sum of electronic and thermal Energies= -243.750431

Sum of electronic and thermal Enthalpies= -243.749487

Sum of electronic and thermal Free Energies= -243.789651

TS4

B3LYP-D3(BJ)/def2-TZVP

0 3

H	-0.72116100	-0.03308300	0.00000700
C	1.29961900	0.02852300	0.00001700
O	2.42009900	-0.01940600	-0.00000900
N	-1.77984700	-0.70005800	-0.00000200
O	-1.74730200	0.61470000	-0.00000200

Zero-point correction= 0.015529

Thermal correction to Energy= 0.021320

Thermal correction to Enthalpy= 0.022264

Thermal correction to Gibbs Free Energy= -0.014980

Sum of electronic and zero-point Energies= -243.794143

Sum of electronic and thermal Energies= -243.788353

Sum of electronic and thermal Enthalpies= -243.787409

Sum of electronic and thermal Free Energies= -243.824653
